# Sex, environment, and death rate in a dementia cohort: a seven-years Bayesian survival analysis using medications data from a contaminated area in Italy

**DOI:** 10.3389/fpubh.2024.1380609

**Published:** 2024-06-11

**Authors:** Antonia Mincuzzi, Paolo Lodeserto, Jennifer Zollino, Rodolfo Sardone, Lucia Bisceglia, Francesco Addabbo, Sante Minerba, Vito Gregorio Colacicco, Orazio Valerio Giannico

**Affiliations:** ^1^Unit of Statistics and Epidemiology, Local Health Authority of Taranto, Taranto, Italy; ^2^Epidemiology and Care Intelligence Area, Strategic Regional Agency for Health and Social Care of Apulia, Bari, Italy; ^3^Healthcare Management, Local Health Authority of Taranto, Taranto, Italy; ^4^General Management, Local Health Authority of Taranto, Taranto, Italy

**Keywords:** dementia, anti-dementia drug, anti-dementia medication, dementia survival, environmental contamination, environmental pollution, dementia epidemiology, sex differences

## Abstract

**Introduction:**

Studies have analyzed the effects of industrial installations on the environment and human health in Taranto, Southern Italy. Literature documented associations between different variables and dementia mortality among both women and men. The present study aims to investigate the associations between sex, environment, age, disease duration, pandemic years, anti-dementia drugs, and death rate.

**Methods:**

Data from the regional medication registry were used. All women and men with an anti-dementia medication between 2015 and 2021 were included and followed-up to 2021. Bayesian mixed effects logistic and Cox regression models with time varying exposures were fitted using integrated nested Laplace approximations and adjusting for patients and therapy characteristics.

**Results:**

A total of 7,961 person-years were observed. Variables associated with lower prevalence of acetylcholinesterase inhibitors (AChEIs) medication were male sex (OR 0.63, 95% CrI 0.42–0.96), age 70–79 years (OR 0.17, 95% CrI 0.06–0.47) and ≥ 80 years (OR 0.08, 95% CrI 0.03–0.23), disease duration of 2–3 years (OR 0.43, 95% CrI 0.32–0.56) and 4–6 years (OR 0.21, 95% CrI 0.13–0.33), and pandemic years 2020 (OR 0.50, 95% CrI 0.37–0.67) and 2021 (OR 0.47, 95% CrI 0.33–0.65). Variables associated with higher mortality were male sex (HR 2.14, 95% CrI 1.75–2.62), residence in the contaminated site of national interest (SIN) (HR 1.25, 95% CrI 1.02–1.53), age ≥ 80 years (HR 6.06, 95% CrI 1.94–18.95), disease duration of 1 year (HR 1.50, 95% CrI 1.12–2.01), 2–3 years (HR 1.90, 95% CrI 1.45–2.48) and 4–6 years (HR 2.21, 95% CrI 1.60–3.07), and pandemic years 2020 (HR 1.38, 95% CrI 1.06–1.80) and 2021 (HR 1.56, 95% CrI 1.21–2.02). Variables associated with lower mortality were therapy with AChEIs alone (HR 0.69, 95% CrI 0.56–0.86) and in combination with memantine (HR 0.54, 95% CrI 0.37–0.81).

**Discussion:**

Male sex, age, disease duration, and pandemic years appeared to be associated with lower AChEIs medications. Male sex, residence in the SIN of Taranto, age, disease duration, and pandemic years seemed to be associated with an increased death rate, while AChEIs medication seemed to be associated with improved survival rate.

## Introduction

Dementia stands as a prominent contributor to global morbidity and mortality, marked by approximately 10 million new cases every year and 55 million existing cases worldwide, with over 60% residing in low-and middle-income nations. Presently, it ranks as the seventh primary cause of death and a significant factor leading to disability and dependency among older populations worldwide. Projections indicate a rise in dementia cases from 57.4 million in 2019 to an estimated 152.8 million by 2050 globally. Alzheimer’s disease accounts for 60–70% of cases, constituting the most frequent form of dementia (1, 2).

Women are disproportionately affected by dementia, with an estimated female-to-male ratio of 1.69 in 2019 (1, 2). In 2021, Italy reported 34,299 dementia deaths (mortality rate per 10,000 inhabitants: 5.79), 23,637 in women (7.78) and 10,662 in men (3.69) (3, 4). On the other hand, results from a cohort study among patients with dementia in Netherlands reported higher mortality risks in men compared to women. Specifically, the study found that the 1-year mortality risk was 30.5% for women and 38.3% for men, while the 5-year mortality risks were 58.5 and 65.4%, respectively (5).

While age represents the primary recognized risk factor for dementia, its onset is not an inevitable consequence of biological aging. Moreover, dementia does not exclusively affect older individuals, as evidenced by the existence of young onset dementia, characterized by symptom manifestation before the age of 65, which accounts for up to 9% of cases. Research indicates that adopting a healthy lifestyle can mitigate the risk of cognitive impairment and dementia. This entails engaging in regular physical activity, refraining from smoking and excessive alcohol consumption, managing weight, controlling blood pressure, cholesterol, and blood sugar levels, and adhering to a nutritious diet. Additional risk factors for cognitive decline and dementia encompass depression, social isolation, limited educational attainment, cognitive inactivity, and exposure to air pollution (1). Evidence suggests that greater exposure to airborne pollutants is linked to an increased risk of developing dementia. Fine particulate matter (PM_2.5_) and nitrogen oxides may pose risks for dementia, though there is more limited data on the latter two (6–8). In general, environmental pressures could have a role in dementia developing. Moderate evidence links the following risk factors: air pollution, aluminum exposure, silicon exposure, selenium levels, vitamin D deficiency, and exposure to pesticide and electro-magnetic fields (9).

A recent meta-analysis suggests that mortality rates among individuals with dementia increased significantly during the COVID-19 (COronaVIrus Disease 19) pandemic, even for those without COVID-19. Specifically, the study found a 25% increase in mortality risk during the period analyzed. These results indicate that individuals with dementia experienced a significant increase in mortality during the pandemic, regardless of their COVID-19 status (10).

In Italy, the medications approved for the treatment of Alzheimer’s disease include acetylcholinesterase inhibitors (donepezil, rivastigmine, and galantamine) and memantine, an (N-methyl-D-aspartate) NMDA receptor antagonist. These drugs are included in the coverage provided by the Italian National Health System. Acetylcholinesterase inhibitors are sanctioned for mild and moderate dementia cases, while memantine is only indicated for moderate forms of the condition (11, 12). Additionally, recent studies have suggested that patients treated with acetylcholinesterase inhibitors may experience cognitive benefits and reduced mortality (11, 13, 14).

In certain regions of the Taranto Province, located in the Apulia Region of Southern Italy, multiple industrial installations and sources of pollution, including a steel plant, an oil refinery, urban discharges, the shipyard of the Italian Navy, and harbor activities have been the subject of extensive research being well-known for their adverse impacts on both the environment and human health (15–30). Regarding environmental, feed, and food impacts, it is crucial to note that the area exhibits contamination of these matrices with metals and persistent organic pollutants, particularly dioxins and PCBs. Furthermore, certain substances have been identified in human biomonitoring studies (17, 20–25).

Regarding human health effects, several evidence has emerged from studies examining populations residing in the contaminated site of national interest (SIN) in Taranto. Cohort studies have highlighted an elevated risk of various cancer incidences (16, 27). Additionally, certain investigations have observed an increased risk of hospitalization for all causes, as well as for circulatory, respiratory, digestive, and urinary diseases (16, 29). Moreover, different studies have pointed to a heightened risk of all-cause mortality, as well as mortality from circulatory and digestive diseases, and various types of cancer (16, 29).

In summary, literature have documented associations between various factors and dementia mortality in both women and men. The present study aims to investigate the associations between sex, environment, age, disease duration, pandemic years, anti-dementia drugs, and death rate.

## Methods

### Study area and baseline epidemiological data

**Figure 1:**
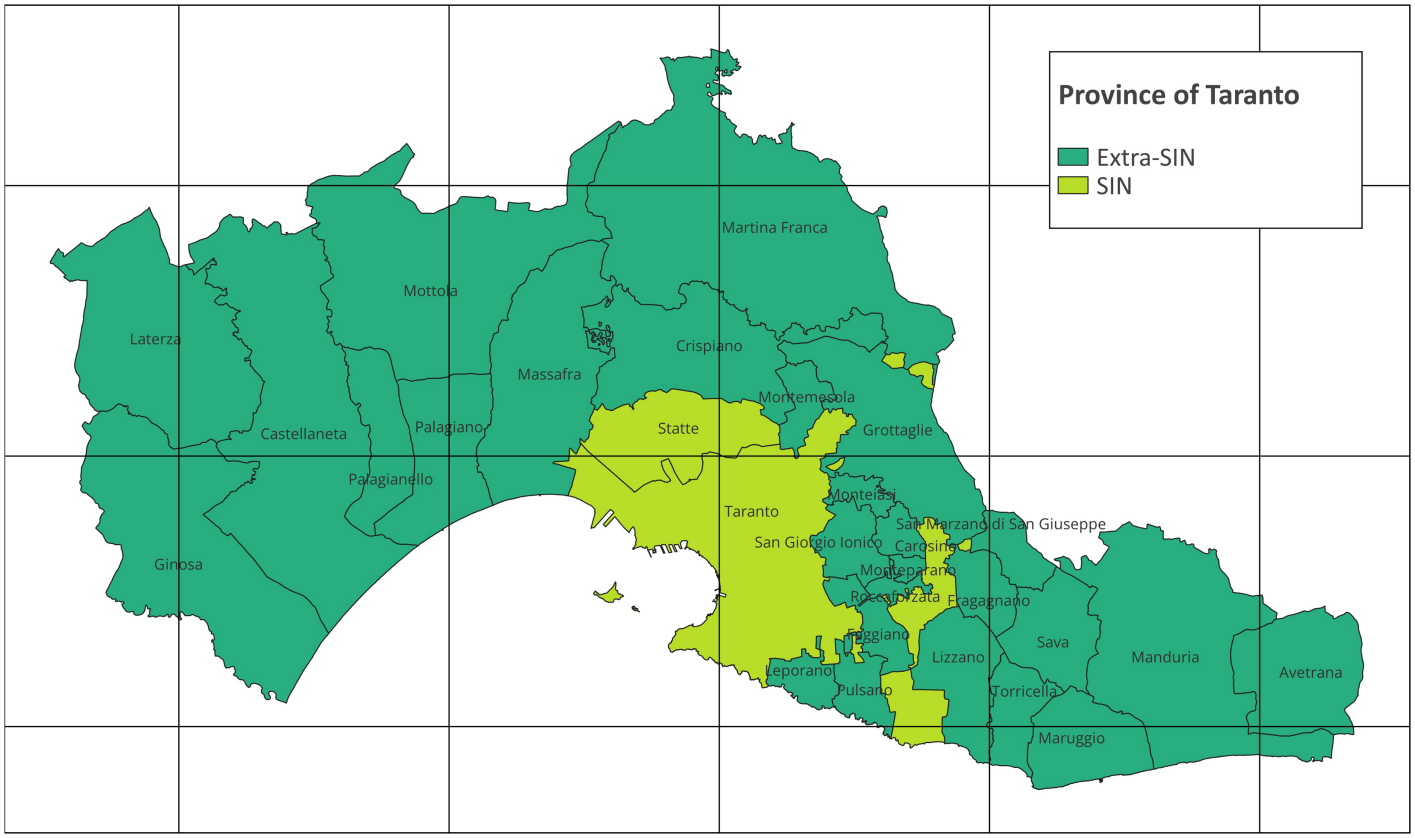
Map of the province of Taranto (grid interval: 20 km; EPSG:32632 – WGS 84 / UTM zone 32 N; Modified from: Italian National Institute of Statistics. Administrative boundaries. https://www.istat.it/it/archivio/222527).

The epidemiologic study focuses on the Taranto Province (Apulia Region, Southern Italy), encompassing 29 municipalities with a combined resident population of 559,892 individuals as of January 1, 2022. Within the province of Taranto there is the SIN of Taranto, composed of the two municipalities of Taranto and Statte. On January 1, 2022, Taranto and Statte had a population of 189,461 and 13,136, respectively (4, 29). The map in Figure 1 represents the study area with municipalities and SIN and was created with QGIS version 3.28.4.

In 2021, Apulia Region reported 2,121 dementia deaths (mortality rate per 10,000 inhabitants: 5.39), 1,384 in women (6.85) and 737 in men (3.85). In the same period, Taranto Province reported 310 dementia deaths (5.52), 189 (6.53) in women and 121 (4.44) in men (3, 4).

### Data source, medications cohort, and inclusion/exclusion criteria

Data collected from the regional medication registry were used to build the cohort. All women and men aged ≥40 with an anti-dementia medication between 01 January 2015 and 31 December 2021 who resided in the Taranto Province at the time of medication were included. Anatomical Therapeutic Chemical (ATC) codes of anti-dementia medications were NO6DA (acetylcholinesterase inhibitors, AChEIs: donepezil, rivastigmine and galantamine), and N06DX (other anti-dementia drugs: memantine, an NMDA receptor antagonist which is the only medication of this group approved in Italy) (11, 12). The follow-up period considered for our cohort was until 31 December 2021. We reduced duplicate medications within each year into a single patient-year observation retaining all available information. To consider the time-varying nature of the analyzed variables on yearly basis, we structured the cohort in observation periods (years) ranging theoretically from 0 to 365 person-days. For each observation period, we assumed an average use of the anti-dementia drug over the year. All variables refer to the specific year. All-cause mortality data relative to the overall follow-up period (2015–2021) were retrieved from the Taranto Province’s Causes of Death Registry, with the information of the day of decease. Patients alive and with no medication to 31/12/2021 probably due to stopping treatment or extra-provincial transfer (right-censoring or interval-censoring, i.e., loss-to-follow-up) contributed to the person-time only for the periods with recorded anti-dementia medications. Patients who already had an anti-dementia medication in 2013 and/or 2014 were excluded from the cohort to exclude prevalent cases. This was the only exclusion criteria in order to build an incident cases cohort. In this way, the first medication in the studied years (2015–2021) was used as a proxy of the disease onset and the starting point to approximate a disease duration (see next sections).

### Study design and variables

This retrospective observational study consists of a cross-sectional study (prevalence study) and a longitudinal study (incidence study with survival analysis) (15, 30). Residence in the SIN was used as an environmental exposures proxy. Age classes were 40–59, 60–69, 70–79, and ≥ 80 years. The difference between an observed year and the first treatment year was assumed as disease duration (0, 1, 2–3 and 4–6 years). To account for the influence of the SARS-CoV-2 pandemic, we categorized treatment years as 2015–19 (non-pandemic years), 2020 and 2021 (pandemic years). Anti-dementia drugs were memantine (NO6DX), acetylcholinesterase inhibitors (NO6DA), and a combination therapy (NO6DA + X).

In the cross-sectional study, the studied outcome was the therapy with memantine or acetylcholinesterase inhibitors (prevalence), and the studied exposures were sex, residence in SIN, age, disease duration and treatment year(s). This study aims to analyze the associations between these factors and the specific therapy. In the longitudinal study, the studied outcome was the all-cause death (incidence), and the studied exposures were sex, residence in SIN, age, disease duration, treatment year(s) and anti-dementia drug(s). This study aims to analyze the associations between these factors and death. To account for the hypothetical heterogeneity related to unobserved ecological or individual level variables, we adjusted for the patient identifier (ID) and municipality of residence (15, 30). Adjustment for the patient ID was also applied to account for the design of the analyses in which the same subject could be observed with different exposures across different time periods.

### Statistical analysis

We conducted data analysis using R version 4.2.3, employing Bayesian inference with package INLA version 22.12.16. The Integrated Nested Laplace Approximation (INLA) method offers a deterministic approach to Bayesian computations applicable to a broad class of models known as Latent Gaussian Models. INLA facilitates swift and precise approximations of posterior marginal by leveraging Laplace approximations and sophisticated numerical techniques, capitalizing on computational efficiencies through sparse matrices. In most scenarios, INLA proves to be both faster and more accurate than alternative methods for Bayesian computation. To address potential non-independence and heterogeneity of observations, mixed models incorporating both fixed and random effects were utilized (15, 30–35).

The cross-sectional study investigated the associations between sex, residence in SIN, age, disease duration, treatment year(s), and therapy with memantine or acetylcholinesterase inhibitors through mixed effects binary logistic regressions. Memantine (N06DX or N06DA + X) or acetylcholinesterase inhibitors therapy (NO6DA or N06DA + X) were the outcome measures binary variables. Sex (Women, men), residence in SIN (Extra SIN, SIN), age class (40–59, 60–69, 70–79, and ≥ 80 years), disease duration (0, 1, 2–3 and 4–6 years), and treatment year(s) (2015–19, 2020, 2021) were included as fixed effects binary or multinominal variables. Patient ID and municipality of residence (random intercepts) were included as random effects multinominal variables. Bayesian binary logistic regression models were fitted calculating odds ratios (OR) and 95% credible intervals (CrI). For patient ID and municipality of residence, an independent and identically distributed random distribution was chosen (15, 30, 32–34).

The longitudinal study investigated the associations between sex, residence in SIN, age, disease duration, treatment year(s), anti-dementia drug(s), and death through a mixed effects cox proportional hazard regression. The difference in days between the 1 January of each year and the last day of follow-up of the considered year (event or right censoring) was the time-axis. All-cause death was the outcome measure binary variable (event). The proportional hazards assumption was tested by analyzing the plotted survival curves between the different levels of the variables. Sex (Women, men), residence in SIN (Extra SIN, SIN), age class (40–59, 60–69, 70–79, and ≥ 80 years), disease duration (0, 1, 2–3 and 4–6 years), treatment year(s) (2015–19, 2020, 2021), and anti-dementia drug(s) (Memantine, AChEIs, combination) were included as fixed effects binary or multinominal variables. Patient ID and municipality of residence (random intercepts) were included as random effects multinominal variables. A Bayesian Cox regression model was fitted calculating hazard ratios (HR) and 95% credible intervals (CrI). For patient ID and municipality of residence, an independent and identically distributed random distribution was chosen. An order two random walk model was chosen for the baseline hazard function (15, 30–35).

Generalized Variance Inflation Factors (GVIF) were calculated to assess multicollinearity within the dataset. Sensitivity analyses were conducted to evaluate the impact of variations in models, methods, variables, and exclusion/inclusion criteria on the results. Various combinations of patients and variables were examined, with exploration of different collapsed categories for included variables and changes in the classification of estimated effects (fixed or random). Additionally, models were repeatedly refitted by excluding each age class and year from the dataset one at a time (15, 30).

Finally, to evaluate possible sex-specific or residence-specific associations, all the analyses were repeated after stratifying the cohort by sex or by residence in SIN into two pairs of subsets.

## Results

Baseline patients and therapy characteristics are shown in Table 1. A total of 7,961 person-years were observed, 3,019 for men, 3,405 for residents in SIN, and 944 for deceased patients, with a median (IQR) age of 78 (73; 82) years. The number of unique patients is 3,201, while the number of unique combinations of patient-year is 8,139.

**Table 1:** Baseline patients and therapy characteristics and follow-up survival status in the anti-dementia drugs cohort, by sex, residence in SIN and survival status.

Baseline patients and therapy characteristics and follow-up survival status	Anti-dementia drugs cohort						
	*N* = 3,201; person-years = 7,961						
	Women	Men	Extra-SIN	SIN	Survived	Deceased	Total
*Anti-dementia drug(s)*							
Memantine [*n* (%)]	874 (44.52%)	594 (47.98%)	896 (48.54%)	572 (42.21%)	1,263 (44.88%)	205 (52.97%)	1,468 (45.86%)
AChEIs [*n* (%)]	1,004 (51.15%)	595 (48.06%)	871 (47.18%)	728 (53.73%)	1,440 (51.17%)	159 (41.09%)	1,599 (49.95%)
Combined [*n* (%)]	85 (4.33%)	49 (3.96%)	79 (4.28%)	55 (4.06%)	111 (3.94%)	23 (5.94%)	134 (4.19%)
*Sex*							
Women [*n* (%)]	1,963 (100.00%)	0 (0.00%)	1,137 (61.59%)	826 (60.96%)	1,788 (63.54%)	175 (45.22%)	1,963 (61.32%)
Men [*n* (%)]	0 (0.00%)	1,238 (100.00%)	709 (38.41%)	529 (39.04%)	1,026 (36.46%)	212 (54.78%)	1,238 (38.68%)
*Residence in SIN*							
Extra-SIN [*n* (%)]	1,137 (57.92%)	709 (57.27%)	1,846 (100.00%)	0 (0.00%)	1,642 (58.35%)	204 (52.71%)	1,846 (57.67%)
SIN [*n* (%)]	826 (42.08%)	529 (42.73%)	0 (0.00%)	1,355 (100.00%)	1,172 (41.65%)	183 (47.29%)	1,355 (42.33%)
*Age (years)*							
Age [Median(IQR)]	79.0 [74.0;83.0]	77.0 [72.0;82.0]	78.0 [73.0;82.0]	78.0 [73.0;82.0]	78.0 [73.0;82.0]	80.0 [76.0;85.0]	78.0 [73.0;82.0]
40–59 [*n* (%)]	53 (2.70%)	47 (3.80%)	48 (2.60%)	52 (3.84%)	95 (3.38%)	5 (1.29%)	100 (3.12%)
60–69 [*n* (%)]	213 (10.85%)	150 (12.12%)	234 (12.68%)	129 (9.52%)	342 (12.15%)	21 (5.43%)	363 (11.34%)
70–79 [*n* (%)]	824 (41.98%)	580 (46.85%)	810 (43.88%)	594 (43.84%)	1,252 (44.49%)	152 (39.28%)	1,404 (43.86%)
≥ 80 [*n* (%)]	873 (44.47%)	461 (37.24%)	754 (40.85%)	580 (42.80%)	1,125 (39.98%)	209 (54.01%)	1,334 (41.67%)
*First treatment year(s)*							
2015 [*n* (%)]	343 (17.47%)	220 (17.77%)	289 (15.66%)	274 (20.22%)	478 (16.99%)	85 (21.96%)	563 (17.59%)
2016 [*n* (%)]	297 (15.13%)	181 (14.62%)	249 (13.49%)	229 (16.90%)	411 (14.61%)	67 (17.31%)	478 (14.93%)
2017 [*n* (%)]	292 (14.88%)	190 (15.35%)	283 (15.33%)	199 (14.69%)	398 (14.14%)	84 (21.71%)	482 (15.06%)
2018 [*n* (%)]	291 (14.82%)	169 (13.65%)	294 (15.93%)	166 (12.25%)	418 (14.85%)	42 (10.85%)	460 (14.37%)
2019 [*n* (%)]	296 (15.08%)	205 (16.56%)	302 (16.36%)	199 (14.69%)	443 (15.74%)	58 (14.99%)	501 (15.65%)
2020 [*n* (%)]	203 (10.34%)	126 (10.18%)	195 (10.56%)	134 (9.89%)	294 (10.45%)	35 (9.04%)	329 (10.28%)
2021 [*n* (%)]	241 (12.28%)	147 (11.87%)	234 (12.68%)	154 (11.37%)	372 (13.22%)	16 (4.13%)	388 (12.12%)
*Survival status at the end of follow-up*							
Survived [*n* (%)]	1,788 (91.09%)	1,026 (82.88%)	1,642 (88.95%)	1,172 (86.49%)	2,814 (100.00%)	0 (0.00%)	2,814 (87.91%)
Deceased [*n* (%)]	175 (8.91%)	212 (17.12%)	204 (11.05%)	183 (13.51%)	0 (0.00%)	387 (100.00%)	387 (12.09%)
Person-years [Sum]	4,942	3,019	4,556	3,405	7,017	944	7,961

Province of Taranto, 2015–21, follow-up to 31-12-2021. Time: days of follow-up. N, cohort.

The results of the binary logistic regression models are shown in Table 2. The variables (fixed effects) associated with higher prevalence of memantine medication were male sex (OR 1.73, 95% CrI 1.13–2.65), age ≥ 80 years (OR 4.55, 95% CrI 1.59–13.09), disease duration of 1 year (OR 4.39, 95% CrI 3.32–5.83), 2–3 years (OR 12.18, 95% CrI 8.73–17.15) and 4–6 years (OR 18.15, 95% CrI 11.05–30.12), and treatment years 2020 (OR 2.00, 95% CrI 1.44–2.76) and 2021 (OR 2.53, 95% CrI 1.76–3.62). The variables (fixed effects) associated with lower prevalence of acetylcholinesterase inhibitors medication were male sex (OR 0.63, 95% CrI 0.42–0.96), age 70–79 years (OR 0.17, 95% CrI 0.06–0.47) and ≥ 80 years (OR 0.08, 95% CrI 0.03–0.23), disease duration of 2–3 years (OR 0.43, 95% CrI 0.32–0.56) and 4–6 years (OR 0.21, 95% CrI 0.13–0.33), and treatment years 2020 (OR 0.50, 95% CrI 0.37–0.67) and 2021 (OR 0.47, 95% CrI 0.33–0.65). Stratified logistic regression models by sex and residence are shown in Supplementary Tables S1–S4. We report here only the associations found in the subsets but not in the full cohort. The variable (fixed effect) associated with lower prevalence of memantine medication in women was residence in SIN (OR 0.25, 95% CrI 0.14–0.42). The variable (fixed effect) associated with higher prevalence of acetylcholinesterase inhibitors medication in women was residence in SIN (OR 3.41, 95% CrI 1.99–5.88). The variables (fixed effects) associated with higher prevalence of acetylcholinesterase inhibitors medication in men were residence in SIN (OR 2.04, 95% CrI 1.04–4.05) and age 60–69 years (OR 5.98, 95% CrI 1.10–32.98).

**Table 2:** Results of mixed effects Bayesian INLA binary logistic regressions models in the anti-dementia drugs cohort, mutually adjusted and adjusted for patient ID and municipality of residence.

Mixed effects INLA binary logistic regressions	Anti-dementia drugs cohort			
	Memantine medication		AChEIs medication	
	*N* = 8,139; *n* = 4,551		*N* = 8,139; *n* = 4,368	
Fixed effects	OR	95% CrI	OR	95% CrI
*Sex*				
Women	1.00	(ref)	1.00	(ref)
Men	1.73	1.13–2.65	0.63	0.42–0.96
*Residence in SIN*				
Extra-SIN	1.00	(ref)	1.00	(ref)
SIN	0.20	0.02–1.90	7.04	0.56–92.28
*Age (years)*				
40–59	1.00	(ref)	1.00	(ref)
60–69	0.48	0.17–1.36	1.21	0.43–3.42
70–79	2.01	0.71–5.69	0.17	0.06–0.47
≥ 80	4.55	1.59–13.09	0.08	0.03–0.23
*Disease duration (years)*				
0	1.00	(ref)	1.00	(ref)
1	4.39	3.32–5.83	0.99	0.77–1.26
2–3	12.18	8.73–17.15	0.43	0.32–0.56
4–6	18.15	11.05–30.12	0.21	0.13–0.33
*Treatment year(s)*				
2015–19	1.00	(ref)	1.00	(ref)
2020	2.00	1.44–2.76	0.50	0.37–0.67
2021	2.53	1.76–3.62	0.47	0.33–0.65

Province of Taranto, 2015–21, follow-up to 31-12-2021. Outcome (prevalence): therapy. N, prevalent cases and non-cases. n, prevalent cases.

**Figure 2:**
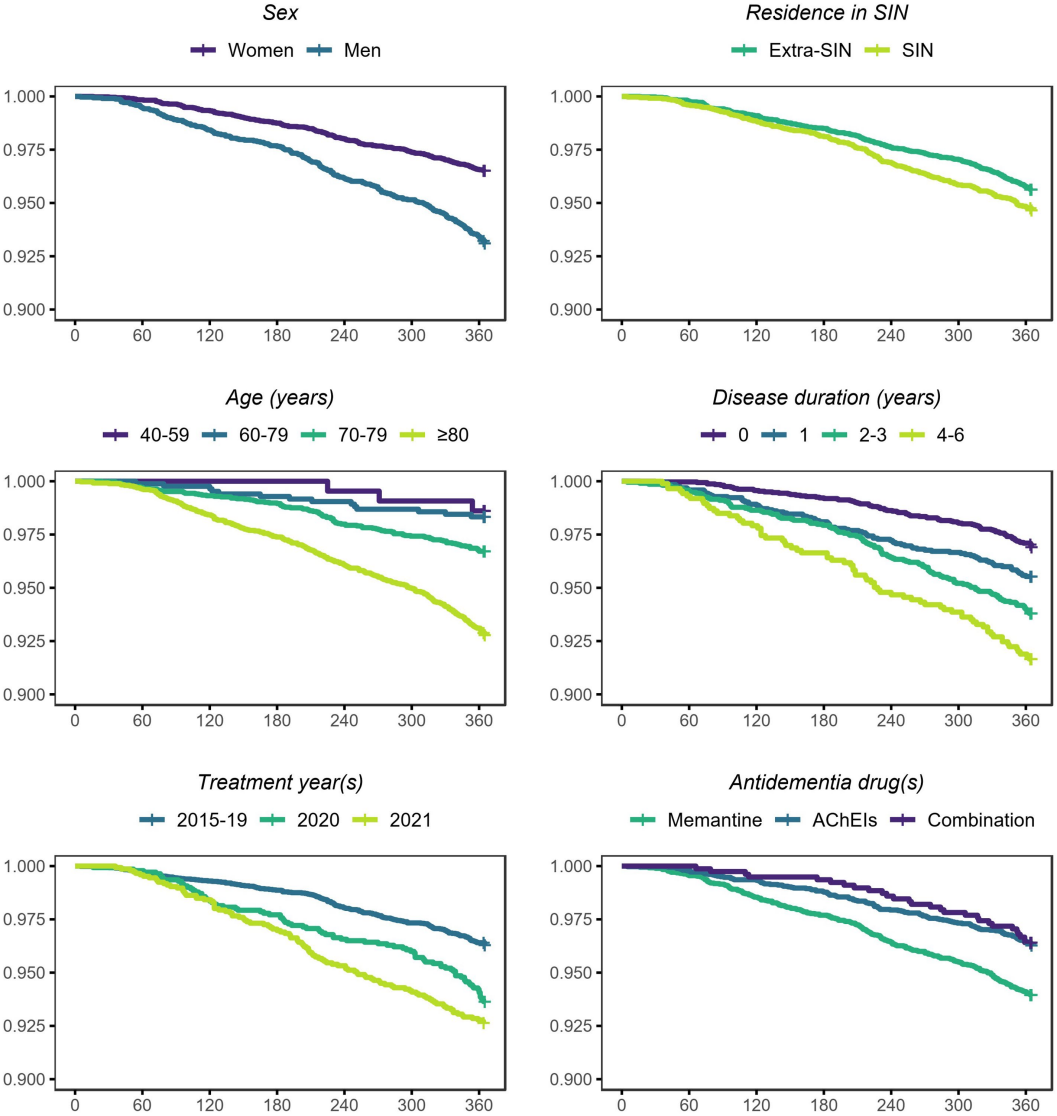
Survival probabilities (y) in the anti-dementia drugs cohort, conditional on each analyzed variable and unconditional on other variables. Province of Taranto, 2015–21, follow-up to 31-12-2021. Time: days of follow-up (x). Outcome (incidence): all-cause death. N: incident cases and non-cases. N: women = 5,026, men = 3,113; extra-SIN = 4,647, SIN = 3,492; age 40–59 = 216, 60–69 = 838, 70–79 = 3,530, ≥80 = 3,555; disease duration 0 = 3,201, 1 = 1946, 2–3 = 2,129, 4–6 = 863; 2015–19 = 5,286, 2020 = 1,399, 2021 = 1,454; memantine = 3,771, AChEIs = 3,588, combination = 780.

Survival probabilities by each analyzed variable are plotted in Figure 2. The curves showed possible associations between survival probability and sex, residence in SIN, age, disease duration, treatment year(s), and anti-dementia drug(s). The results of the Cox proportional hazard regression model are shown in Table 3. The variables (fixed effects) associated with higher death rate were male sex (HR 2.14, 95% CrI 1.75–2.62), residence in SIN (HR 1.25, 95% CrI 1.02–1.53), age ≥ 80 years (HR 6.06, 95% CrI 1.94–18.95), disease duration of 1 year (HR 1.50, 95% CrI 1.12–2.01), 2–3 years (HR 1.90, 95% CrI 1.45–2.48) and 4–6 years (HR 2.21, 95% CrI 1.60–3.07), and treatment years 2020 (HR 1.38, 95% CrI 1.06–1.80) and 2021 (HR 1.56, 95% CrI 1.21–2.02). The variables (fixed effects) associated with lower death rate were therapy with acetylcholinesterase inhibitors alone (HR 0.69, 95% CrI 0.56–0.86) and in combination with memantine (HR 0.54, 95% CrI 0.37–0.81). Stratified Cox proportional hazard regression models by sex and residence are shown in Supplementary Tables S5–S8. We report here only the associations found in the subsets but not in the full cohort. The variable (fixed effect) associated with higher death rate in men was age 70–79 years (HR 8.40, 95% CrI 1.18–60.04).

**Table 3:** Results of mixed effects Bayesian INLA Cox proportional hazard regression model in the anti-dementia drugs cohort, mutually adjusted and adjusted for patient ID and municipality of residence.

Mixed effects INLA Cox proportional hazard regression	Anti-dementia drugs cohort	
	All-cause death	
	*N* = 8,139; person-years = 7,961; *n* = 387	
Fixed effects	HR	95% CrI
*Sex*		
Women	1.00	(ref)
Men	2.14	1.75–2.62
*Residence in SIN*		
Extra-SIN	1.00	(ref)
SIN	1.25	1.02–1.53
*Age (years)*		
40–59	1.00	(ref)
60–69	1.50	0.43–5.24
70–79	2.78	0.88–8.76
≥ 80	6.06	1.94–18.95
*Disease duration (years)*		
0	1.00	(ref)
1	1.50	1.12–2.01
2–3	1.90	1.45–2.48
4–6	2.21	1.60–3.07
*Treatment year(s)*		
2015–19	1.00	(ref)
2020	1.38	1.06–1.80
2021	1.56	1.21–2.02
*Anti-dementia drug(s)*		
Memantine	1.00	(ref)
AChEIs	0.69	0.56–0.86
Combined	0.54	0.37–0.81

Province of Taranto, 2015–21, follow-up to 31-12-2021. Time: days of follow-up. Outcome (incidence): all-cause death. N, incident cases and non-cases. n, incident cases.

## Discussion

The results of our study suggest that sex, environment, age, disease duration, pandemic years, and type of medication are independent prognostic factor in patients in treatment with anti-dementia drugs. In our cohort, therapy with AChEIs seemed to be associated with lower mortality compared to therapy with memantine alone. This result is also consistent with findings from other studies (11, 13, 14).

Sex, age, disease duration and pandemic years also appeared to be associated with the choice of anti-dementia drug. In this regard, an interesting finding is the lower prevalence of medications for acetylcholinesterase inhibitors (alone or in combination) in patients with a longer duration of the disease compared to memantine alone. This could be explained by the difference in recommendations between the two classes of medications available in Italy, as memantine is only recommended in moderate forms of dementia. Disease duration is therefore a kind of proxy for the unobserved variable of disease severity. Disease duration is also independently associated with a higher mortality rate, thus maybe exerting a direct and indirect - through lower AChEI medication - effect on mortality. Similar results were found for male sex, older age, and pandemic years which are associated with higher mortality and with lower prevalence of AChEIs medication. The results about pandemic years and medications are intriguing. Based on our data it seems that during the COVID-19 pandemic the dementia therapy had switched from AChEIs to memantine. Moreover, the absolute number of antidementia medications dropped in 2020. We can hypothesize these differences to be related to changes in the clinical presentation of the disease or to limited accessibility of health services (10, 11). As expected, in 2020–21 we found a higher mortality among individuals with dementia. Shifting the focus of care toward acute COVID-19 related treatment could impact the attention and resources allocated to chronic and non-communicable diseases. Pandemic determined challenges in providing adequate medical care to those with dementia. Moreover, isolation adversely impacts the well-being of older individuals and exacerbates symptoms in those with dementia. Limited access to physical activity and mental stimulation may potentially contribute to the physical decline of individuals with dementia. Individuals residing in care facilities who have dementia have encountered different degrees of social isolation and a reduction in mental stimulation. Furthermore, senior individuals residing in their own homes who suffer from dementia, together with their caregivers, have reported experiencing a decline in their daily routines, feelings of isolation, and difficulties in obtaining regular healthcare services (10).

Regarding sex disparities in dementia prognosis, a noteworthy finding suggests a correlation between male sex and a higher all-cause death rate. This observation was observed even after adjusting the hazard ratio (HR) for other variables considered in the Bayesian mixed effects regression model. This result appears to align with sex-based distinctions in dementia prognosis documented in existing literature (5).

Namely, this means that men in our dementia cohort have an adjusted excess relative risk for all-cause death of 114% (95% CrI 75–162%) compared to women. Moreover, men also present a lower prevalence of AChEIs medication, namely a negative excess odds ratio for AChEIs medication of 37% (95% CrI 4–58%) compared to women, which is indirectly related to increased all-cause death rate as well. Additionally, this is consistent (in effect direction) with the findings reported in a study on lung cancer patients in the province of Taranto, which showed in male patients (compared to female patients) an excess relative risk for all-cause death of 24% (95% CrI 7–43%) (30).

In general, differences between sexes have been noted among dementia patients concerning risk factor profiles, symptomatology, biomarkers, disease progression, and treatment. Accumulating evidence underscores sex-specific patterns in how the disease manifests, as well as disparities in rates of cognitive impairment and brain atrophy, suggesting that sex plays a pivotal role in disease heterogeneity. Emerging insights into sex-related variances in neural anatomy and function suggest that sex could play a crucial role in categorizing patients with Alzheimer’s disease and tailoring personalized treatment approaches. Despite the current intense scrutiny of sex’s impact on dementia epidemiology, the concept of sex-specific clinicopathological phenotypes of Alzheimer’s disease remains largely unexplored (36–38).

In relation to the influence of environmental pressures on dementia, this study identified in women and men an association between residing in the SIN and an higher frequency of acetylcholinesterase inhibitors medications. This finding is interesting and may be related to differences in both disease presentation and treatment due to environmental or socioeconomic factors. However, the most interesting finding could be the increased all-cause death rate associated to the residence in the SIN. This outcome was noted irrespective of all other variables examined, as the hazard ratio (HR) was adjusted for the additional factors incorporated in the Bayesian mixed effects regression model. To summarize, compared to the residents in the other municipalities of the province, it seems that the subjects in our cohort which resided in the SIN have an adjusted excess relative risk for all-cause mortality of 25% (95% CrI 2–53%).

This excess mortality could be linked to environmental factors possibly associated with the documented anthropic pressures in the SIN: steel plant, oil refinery, harbor activities, etc. (29). In fact, difference studies have reported for this area a contamination of environmental, feed, and food matrices (e.g., mussels, eggs) with persistent organic pollutants (dioxins and polychlorinated biphenyls) and metals, possibly resulting in foodborne exposure. Some of these substances or their metabolites/markers have also been detected in human biomonitoring studies (17, 20–25). Concerning air pollution, industrial-originated air pollution (e.g., polycyclic aromatic hydrocarbons, sulfur dioxide, particulate matter) and health impacts have been documented in the area (15, 16, 18, 19, 26, 28, 29, 39).

Our results validate existing knowledge concerning the higher risk of all-cause mortality among both men and women residing in the SIN of Taranto. Nevertheless, our findings also indicate that the excess relative risk within the analyzed cohort may be more pronounced, as recent study for the years 2013–17 focused on the broader population living in the area have revealed an excess relative risk for all-cause death of 7% (90% CI 5–9%) in women and of 10% (90% CI 8–13%) in men in the SIN of Taranto (reference: Apulia Region) (29). While we are examining diverse periods and methodologies, and the credible interval of the present study entirely encompasses the mentioned estimates along with their confidence intervals, we cannot dismiss the possibility that the excess mortality risk in the SIN of Taranto might be different in the cohort of women and men in treatment with anti-dementia drugs. Consequently, our findings could imply that frail dementia patients may exhibit increased vulnerability to the risks linked with a disadvantaged or polluted external environment. This could be also consistent with the similar findings reported in a study on female breast cancer patients in the same area, which showed in SIN (compared to extra-SIN) an excess relative risk for all-cause death of 22% (95% CrI 1–48%) (15).

If verified, these findings pose potential ethical concerns. Indeed, numerous epidemiological studies have previously highlighted an increased risk for various cancer incidences, multiple causes of hospitalization, and all-cause mortality among the general population residing in the SIN of Taranto (15, 16, 19, 26, 27, 29). According to the current study, this excess relative risk for all-cause mortality might be amplified within the cohort of dementia patients.

However, as the present study utilized the residence in SIN (contextual-level variable) to determine exposure to environmental pressures, there is a potential susceptibility to ecological fallacy. Additionally, the exposure assessment lacks specificity as various pollutants could originate from different sources and vary within the studied region (15, 30). Another limitation of the study may be the absence of data concerning hereditary factors and genetics, which unfortunately are not recorded in medication registries or health records. Nevertheless, some influence of these factors might have been indirectly included in the analysis using mixed models with random effects, which incorporate the heterogeneity between patients and areas (15, 30).

Nevertheless, the abundance of existing evidence regarding Taranto lends significant prior plausibility to the association between residence in SIN and the observed increase in mortality in this study. This association was observed despite adjustments for all other included patient and therapy characteristics. Furthermore, aside from the well-documented environmental pressures, the municipalities of Taranto and Statte exhibit relatively high municipal-level deprivation indexes (40). These indexes are regionally referenced and utilized individual data from the general population and housing census of 2011. Five conditions were selected to summarize in the index the multidimensional concept of material and social deprivation: low education levels, unemployment, renting, living in crowded conditions, and living in single-parent families (40). However, caution is warranted in interpreting this index, both due to its ecological-level nature and because the latest index was based on the 2011 census. Therefore, the available deprivation index (40) may not accurately reflect individual-level deprivation during the years covered by our study. Moreover, incorporating the municipality of residence as a random effect in the regression models has somehow offered some adjustment for the socio-economic differences at the municipality level (15, 30, 40).

However, it is crucial to acknowledge that deprivation, socio-economic status, and inequalities may not only influence harmful lifestyle habits (e.g., smoking), health conditions, and mortality rates but could also potentially impact the utilization of healthcare services (40, 41). Additionally, concerning this matter, the SIN largely corresponds to the provincial capital Taranto, which could potentially affect access to healthcare services at both the territorial and hospital levels, as well as in terms of regional and extra-regional mobility. This relates to the problem of the lack of data regarding the diagnostic paths and treatments of these patients, including information about their access to dedicated healthcare services, which is another limitation of the study. However, to this regard we expected low risk of bias, given that the entire studied cohort could theoretically access the same healthcare services since both SIN and extra-SIN areas belong to the same Local Health Authority (15, 30).

Another related concern could arise in relation to the lack of information about the presence of comorbidities in these patients. In fact, as expected our cohort is largely made up of elder people, who are typically affected by multiple conditions. Unfortunately, we do not have this piece of information in the used data source to fill this gap. Nevertheless, this lack of data is not supposed to compromise the validity of the inference about the influence of sex and environment on survival. Namely, in the cloud of all the reasonable causal diagrams which could describe the associations between these variables and mortality, surely comorbidities cannot be determinants of sex and environmental pressures. Therefore, they could not be confounders, but rather mediators in the causal paths. So do not include these factors in the analyses presumably led only to a lack of knowledge about the specific mediators of this associations. Similar considerations can be made about the lack of information about the specific causes of deaths or about lifestyle habits (e.g., sedentary behavior, smoking, alcohol consumption, poor diet quality). In any case, the pre-existing evidence in the SIN about the increased mortality from circulatory causes and various types of cancer could suggest these specific diseases to be involved in the genesis of the observed reduction of survival (16, 29).

Finally, it is worth to discuss the probable most important limitation of the present study, which is the incomplete inclusion of the true dementia-cohort and the potential for sampling and selection bias. These limitations could be associated with the use of a cohort that includes only patients with an anti-dementia medication, but not all patients with a diagnosed dementia disease. Unfortunately, this information is not currently available in healthcare records, nor are data on the specific dementia disease (Alzheimer’s disease or other forms), the severity of the disease (Mini-Mental State Examination score or other measures of cognitive or general performance status in these patients), the specific time of disease onset (to accurately calculate disease duration), the specific number and timing of medications within the year (to accurately calculate treatment duration for each medication), or the effective adherence or compliance with the therapy. Therefore, we cannot be sure that the associations found in this study are free of bias and generalizable to all people with dementia. However, with these limitations in mind, our results are certainly of interest given the peculiar socio-environmental context, the large number of person-time followed-up, the time-varying exposures, and the multiple factors analyzed.

In summary, as previously mentioned, the absence of individual-level clinical data, heredity and genetics, socio-cultural variables, environmental factors, harmful habits, and healthcare service utilization represents limitations of the current study (15, 30). However, viewed from another perspective, these same elements could serve as starting points for future research endeavors. It would be compelling to enhance and extend this study by acquiring additional individual-level data that are currently unavailable, including clinical details (dementia type, mini-mental state evaluation, other metrics), genetics and hereditary factors, environmental exposures (proximity to polluting sources, airborne pollutant exposure through dispersion models, biomonitoring), socio-economic factors (marital status in conjunction with educational level and gender (42), updated deprivation indicators at ecological or individual level), and healthcare service access (timing, location, and type of interventions).

In conclusion, despite the limitations discussed above, the results showed interesting associations between the analyzed variables. Male sex, age, disease duration, and pandemic years appeared to be associated with lower anticholinesterases medications. Male sex, residence in the SIN of Taranto, age, disease duration, and pandemic years seemed to be associated with an increased death rate, while acetylcholinesterase inhibitors medication appeared to be associated with improved survival.

## Data availability statement

The data analyzed in this study is subject to the following licenses/restrictions: the original contributions presented in the study are included in the article/Supplementary material, further inquiries can be directed to the corresponding author. Requests to access these datasets should be directed to epistat@asl.taranto.it.

## Ethics statement

Ethical approval was not required for the study involving humans in accordance with the local legislation and institutional requirements. Written informed consent to participate in this study was not required from the participants or the participants’ legal guardians/next of kin in accordance with the national legislation and the institutional requirements.

## Author contributions

AM: Writing – review & editing, Supervision, Resources, Project administration, Funding acquisition, Data curation. PL: Writing – review & editing, Software, Methodology, Data curation. JZ: Writing – review & editing, Methodology, Data curation. RS: Writing – review & editing, Visualization, Validation, Supervision, Methodology, Investigation, Funding acquisition, Conceptualization. LB: Writing – review & editing, Validation, Supervision. FA: Writing – review & editing, Validation, Supervision, Investigation. SM: Writing – review & editing, Validation, Supervision, Resources, Project administration, Funding acquisition. VGC: Writing – review & editing, Validation, Supervision, Resources, Project administration, Funding acquisition. OVG: Writing – review & editing, Writing – original draft, Visualization, Validation, Supervision, Resources, Project administration, Methodology, Investigation, Funding acquisition, Formal analysis, Data curation, Conceptualization.
