# Supplementary material for: Sex, environment, and death rate in a dementia cohort: a seven-years Bayesian survival analysis using medications data from a contaminated area in Italy
Source: Front Public Health. 2024 Jun 11;12:1380609. doi: 10.3389/fpubh.2024.1380609 (PMC11216074; doi:10.3389/fpubh.2024.1380609)
Supplement: Supplementary file 1 [file Data_Sheet_1.pdf]

Supplementary materials

Sex, environment, and death rate in a dementia cohort: a seven-years Bayesian survival analysis using medications data from a contaminated area in Italy

Antonia MINCUZZI[1]. Paolo LODESERTO[1]. Jennifer ZOLLINO[1]. Rodolfo SARDONE[1]. Lucia BISCEGLIA[2]. Francesco ADDABBO[1]. Sante MINERBA[3]. Vito Gregorio COLACICCO[4]. Orazio Valerio GIANNICO[1]

1. Unit of Statistics and Epidemiology, Local Health Authority of Taranto, Taranto. Italy
2. Strategic Regional Agency for Health and Social Care of Apulia, Bari, Italy
3. Healthcare Management, Local Health Authority of Taranto, Taranto, Italy
4. General Management, Local Health Authority of Taranto, Taranto, Italy

Corresponding author
Orazio Valerio Giannico
Viale Virgilio 31. 74121. Taranto
oraziovaleriogiannico@gmail.com

Mixed effects INLA binary logistic regressions	***Anti-dementia drugs cohort, women***			
	Memantine medication		***AChEIs medication***	
	N = 5,026; *n* = 2,757		*N* = 5,026; *n* = 2,742	
**Fixed effects**	**OR**	**95% CrI**	**OR**	**95% CrI**
**Residence in SIN**				
Extra-SIN	1.00	(ref)	1.00	(ref)
SIN	0.25	0.14-0.42	3.41	1.99-5.88
**Age (years)**				
40-59	1.00	(ref)	1.00	(ref)
60-69	0.75	0.19-3.02	0.42	0.10-1.73
70-79	2.03	0.50-8.20	0.09	0.02-0.38
≥ 80	5.54	1.36-22.82	0.03	0.01-0.15
**Disease duration (years)**				
0	1.00	(ref)	1.00	(ref)
1	4.37	3.09-6.18	0.88	0.64-1.20
2-3	11.99	7.92-18.05	0.39	0.27-0.55
4-6	19.41	10.51-35.93	0.15	0.08-0.27
**Treatment year(s)**				
2015-19	1.00	(ref)	1.00	(ref)
2020	2.11	1.39-3.21	0.45	0.31-0.67
2021	2.56	1.62-4.05	0.44	0.28-0.67

Table S1. Results of mixed effects Bayesian INLA binary logistic regressions models in the anti-dementia drugs cohort of women, mutually adjusted and adjusted for patient ID and municipality of residence. Province of Taranto, 2015-21, follow-up to 31/12/2021. Outcome (prevalence): therapy. N: prevalent cases and non-cases. n: prevalent cases.

Mixed effects INLA binary logistic regressions	*Anti-dementia drugs cohort, men*			
	Memantine medication		*AChEIs medication*	
	N = 3,113; *n* = 1,794		*N* = 3,113; *n* = 1,626	
**Fixed effects**	**OR**	**95% CrI**	**OR**	**95% CrI**
**Residence in SIN**				
Extra-SIN	1.00	(ref)	1.00	(ref)
SIN	0.75	0.08-6.75	2.04	1.04-4.05
**Age (years)**				
40-59	1.00	(ref)	1.00	(ref)
60-69	0.21	0.04-1.14	5.98	1.10-32.98
70-79	1.89	0.37-9.62	0.36	0.07-1.81
≥ 80	3.04	0.59-15.87	0.25	0.05-1.31
**Disease duration (years)**				
0	1.00	(ref)	1.00	(ref)
1	4.37	2.81-7.05	1.15	0.78-1.71
2-3	12.38	7.27-22.44	0.46	0.29-0.72
4-6	16.74	7.34-40.18	0.35	0.16-0.73
**Treatment year(s)**				
2015-19	1.00	(ref)	1.00	(ref)
2020	1.76	1.05-2.96	0.62	0.38-1.02
2021	2.48	1.38-4.47	0.56	0.32-0.97

Table S2. Results of mixed effects Bayesian INLA binary logistic regressions models in the anti-dementia drugs cohort of men, mutually adjusted and adjusted for patient ID and municipality of residence. Province of Taranto, 2015-21, follow-up to 31/12/2021. Outcome (prevalence): therapy. N: prevalent cases and non-cases. n: prevalent cases.

Mixed effects INLA binary logistic regressions	*Anti-dementia drugs cohort, extra-SIN*			
	Memantine medication		***AChEIs medication***	
	N = 4,647; *n* = 2,733		*N* = 4,647; *n* = 2,335	
**Fixed effects**	**OR**	**95% CrI**	**OR**	**95% CrI**
**Sex**				
Women	1.00	(ref)	1.00	(ref)
Men	1.03	0.59-1.78	0.84	0.48-1.46
**Age (years)**				
40-59	1.00	(ref)	1.00	(ref)
60-69	0.76	0.18-3.29	0.32	0.07-1.57
70-79	3.71	0.85-16.15	0.04	0.01-0.19
≥ 80	12.96	2.92-58.24	0.01	0.00-0.05
**Disease duration (years)**				
0	1.00	(ref)	1.00	(ref)
1	3.88	2.73-5.61	1.05	0.76-1.46
2-3	12.29	8.03-19.49	0.37	0.25-0.54
4-6	20.91	10.85-41.41	0.12	0.06-0.22
**Treatment year(s)**				
2015-19	1.00	(ref)	1.00	(ref)
2020	1.59	1.05-2.42	0.60	0.40-0.90
2021	1.96	1.23-3.14	0.63	0.40-0.99

Table S3. Results of mixed effects Bayesian INLA binary logistic regressions models in the anti-dementia drugs cohort of residents in extra-SIN municipalities, mutually adjusted and adjusted for patient ID and municipality of residence. Province of Taranto, 2015-21, follow-up to 31/12/2021. Outcome (prevalence): therapy. N: prevalent cases and non-cases. n: prevalent cases.

Mixed effects INLA binary logistic regressions	***Anti-dementia drugs cohort, SIN***			
	Memantine medication		***AChEIs medication***	
	N = 3,492; *n* = 1,818		*N* = 3,492; *n* = 2,033	
**Fixed effects**	**OR**	**95% CrI**	**OR**	**95% CrI**
**Sex**				
Women	1.00	(ref)	1.00	(ref)
Men	3.30	1.70-6.53	0.45	0.24-0.85
**Age (years)**				
40-59	1.00	(ref)	1.00	(ref)
60-69	0.40	0.09-1.85	2.60	0.61-11.18
70-79	1.14	0.26-5.07	0.52	0.12-2.14
≥ 80	1.55	0.35-6.99	0.46	0.11-1.93
**Disease duration (years)**				
0	1.00	(ref)	1.00	(ref)
1	5.01	3.26-7.82	0.91	0.62-1.31
2-3	11.57	7.03-19.5	0.51	0.34-0.78
4-6	14.47	6.90-31.19	0.41	0.21-0.78
**Treatment year(s)**				
2015-19	1.00	(ref)	1.00	(ref)
2020	2.67	1.60-4.45	0.39	0.25-0.62
2021	3.49	2.00-6.14	0.32	0.19-0.53

Table S4. Results of mixed effects Bayesian INLA binary logistic regressions models in the anti-dementia drugs cohort of residents in SIN municipalities, mutually adjusted and adjusted for patient ID and municipality of residence. Province of Taranto, 2015-21, follow-up to 31/12/2021. Outcome (prevalence): therapy. N: prevalent cases and non-cases. n: prevalent cases.

Mixed effects INLA<br>*Cox proportional hazard regression*	***Anti-dementia drugs cohort, women***	
	All-cause death	
	N = 5,026; person-years = 4,942; *n* = 175	
**Fixed effects**	**HR**	**95% CrI**
**Residence in SIN**		
Extra-SIN	1.00	(ref)
SIN	1.49	1.10-2.01
**Age (years)**		
40-59	1.00	(ref)
60-69	0.71	0.14-3.52
70-79	1.24	0.30-5.15
≥ 80	3.33	0.82-13.52
**Disease duration (years)**		
0	1.00	(ref)
1	1.40	0.90-2.18
2-3	1.89	1.27-2.81
4-6	2.12	1.31-3.42
**Treatment year(s)**		
2015-19	1.00	(ref)
2020	1.54	1.06-2.25
2021	1.33	0.90-1.97
**Anti-dementia drug(s)**		
Memantine	1.00	(ref)
AChEIs	0.73	0.53-1.01
Combined	0.49	0.26-0.92

Table S5. Results of mixed effects Bayesian INLA Cox proportional hazard regression model in the anti-dementia drugs cohort of women, mutually adjusted and adjusted for patient ID and municipality of residence. Province of Taranto, 2015-21, follow-up to 31/12/2021. Time: days of follow-up. Outcome (incidence): all-cause death. N: incident cases and non-cases. n: incident cases.

Mixed effects INLA<br>*Cox proportional hazard regression*	***Anti-dementia drugs cohort, men***	
	All-cause death	
	N = 3,113; person-years = 3,019; *n* = 212	
**Fixed effects**	**HR**	**95% CrI**
**Residence in SIN**		
Extra-SIN	1.00	(ref)
SIN	1.08	0.82-1.41
**Age (years)**		
40-59	1.00	(ref)
60-69	4.22	0.53-33.58
70-79	8.40	1.18-60.04
≥ 80	15.86	2.23-112.85
**Disease duration (years)**		
0	1.00	(ref)
1	1.59	1.08-2.36
2-3	1.90	1.32-2.75
4-6	2.33	1.49-3.65
**Treatment year(s)**		
2015-19	1.00	(ref)
2020	1.24	0.86-1.79
2021	1.75	1.26-2.45
**Anti-dementia drug(s)**		
Memantine	1.00	(ref)
AChEIs	0.65	0.48-0.88
Combined	0.57	0.34-0.94

Table S6. Results of mixed effects Bayesian INLA Cox proportional hazard regression model in the anti-dementia drugs cohort of men, mutually adjusted and adjusted for patient ID and municipality of residence. Province of Taranto, 2015-21, follow-up to 31/12/2021. Time: days of follow-up. Outcome (incidence): all-cause death. N: incident cases and non-cases. n: incident cases.

Mixed effects INLA<br>*Cox proportional hazard regression*	***Anti-dementia drugs cohort, extra-SIN***	
	All-cause death	
	N = 4,647; person-years = 4,556; *n* = 204	
**Fixed effects**	**HR**	**95% CrI**
**Sex**		
Women	1.00	(ref)
Men	2.49	1.88-3.29
**Age (years)**		
40-59	1.00	(ref)
60-69	2.81	0.36-22.07
70-79	5.12	0.71-36.75
≥ 80	9.11	1.27-65.19
**Disease duration (years)**		
0	1.00	(ref)
1	1.47	0.99-2.19
2-3	1.84	1.28-2.66
4-6	2.13	1.35-3.38
**Treatment year(s)**		
2015-19	1.00	(ref)
2020	1.35	0.94-1.95
2021	1.65	1.17-2.33
**Anti-dementia drug(s)**		
Memantine	1.00	(ref)
AChEIs	0.78	0.57-1.05
Combined	0.58	0.33-1.03

Table S7. Results of mixed effects Bayesian INLA Cox proportional hazard regression model in the anti-dementia drugs cohort of residents in extra-SIN municipalities, mutually adjusted and adjusted for patient ID and municipality of residence. Province of Taranto, 2015-21, follow-up to 31/12/2021. Time: days of follow-up. Outcome (incidence): all-cause death. N: incident cases and non-cases. n: incident cases.

Mixed effects INLA<br>*Cox proportional hazard regression*	***Anti-dementia drugs cohort, SIN***<br>***All-cause death***<br>*N* = 3,492; person-years = 3,405; *n* = 183	
**Fixed effects**	**HR**	**95% CrI**
**Sex**		
Women	1.00	(ref)
Men	1.82	1.36-2.43
**Age (years)**		
40-59	1.00	(ref)
60-69	1.08	0.21-5.59
70-79	2.35	0.57-9.67
≥ 80	6.52	1.61-26.37
**Disease duration (years)**		
0	1.00	(ref)
1	1.54	1.00-2.36
2-3	2.01	1.36-2.99
4-6	2.37	1.48-3.81
**Treatment year(s)**		
2015-19	1.00	(ref)
2020	1.40	0.96-2.04
2021	1.44	0.98-2.10
**Anti-dementia drug(s)**		
Memantine	1.00	(ref)
AChEIs	0.59	0.43-0.81
Combined	0.48	0.28-0.84

Table S8. Results of mixed effects Bayesian INLA Cox proportional hazard regression model in the anti-dementia drugs cohort of residents in SIN municipalities, mutually adjusted and adjusted for patient ID and municipality of residence. Province of Taranto, 2015-21, follow-up to 31/12/2021. Time: days of follow-up. Outcome (incidence): all-cause death. N: incident cases and non-cases. n: incident cases.
